# Melatonin Inhibits Formation of Mitochondrial Permeability Transition Pores and Improves Oxidative Phosphorylation of Frozen-Thawed Ram Sperm

**DOI:** 10.3389/fendo.2019.00896

**Published:** 2020-01-08

**Authors:** Yi Fang, Chengzhen Zhao, Hai Xiang, Xueli Zhao, Rongzhen Zhong

**Affiliations:** Jilin Provincial Key Laboratory of Grassland Farming, Northeast Institute of Geography and Agoecology, Chinese Academy of Sciences, Changchun, China

**Keywords:** ram, sperm, cryopreservation, melatonin, mitochondrial permeability transition pore, oxidative phosphorylation

## Abstract

Structural and functional damages to mitochondria of frozen-thawed sperm are a typical cryoinjury, with mitochondrial permeability transition pore (MPTP) formation being the hallmark change. Mitochondria are both a primary synthesis site and principle target for melatonin; this compound can directly inhibit MPTP formation and therefore confer protection at a mitochondrial level. The objective was to determine effects of melatonin on MPTP opening, viability, motility, and oxidative phosphorylation (OXPHOS) of frozen-thawed ram sperm. Ram semen was diluted in glucose-egg yolk buffer with 0 or 10^−7^ M melatonin (frozen and frozen + melatonin groups, respectively) and slow frozen, with fresh semen as Control. In frozen-thawed sperm, melatonin inhibited MPTP opening and lactate concentrations and improved sperm viability, motility, acetyl-CoA concentration and adenosine triphosphate (ATP) production. With regard to the underlying physiological mechanism, melatonin suppressed movement of citrate synthase, isocitrate dehydrogenase, oxoglutarate dehydrogenase complex, and F0F1-ATP synthase permeability from mitochondrial to cytosolic fractions induced by MPTP opening; furthermore, it increased mRNA expressions of respiratory chain complex components and activities of complexes I, II, III, and IV and thereby improved oxygen consumption capacity in frozen-thawed sperm. In conclusion, melatonin improved OXPHOS of frozen-thawed ram sperm, attributed to inhibition of cryopreservation-induced MPTP opening.

## Introduction

Cryopreserved semen is a common method for preserving and transporting genetic material in most domestic species (1). However, in sheep, <1% of semen is frozen (2), due to extensive structural and functional damage during freezing and thawing reducing sperm quality and fertility (3). Cryopreservation-induced damage to ovine sperm mitochondria reduces energy production and consequently fertility, as the cervix becomes a formidable barrier to sperm transport and sperm have shortened duration of survival in the female reproductive tract (4). However, melatonin protects mitochondria by reducing oxidative stress and preventing mitochondrial permeability transition pore (MPTP) opening (5).

Cryopreservation damages sperm mitochondria, inhibiting metabolism. Based on metabolomic and proteomic analyses, 63% of differentially expressed proteins in sperm with different cryo-tolerance and 23% of differentially expressed proteins between fresh and frozen sperm were associated with regulation of metabolism (6, 7). Furthermore, degradation or loss of the most important metabolism regulatory factors were major cryopreservation-induced injuries, including F1F0-ATP synthase, G protein regulatory inductor 1 of axon and malic dehydrogenase (8). Moreover, most sub-lethal damage was attributed to altered mitochondrial functions, with activation of MPTP having an important role (9).

The MPTP is a multi-component protein aggregate in mitochondria, involving both inner and outer mitochondrial membranes. This complex has two functions: integration of oxidative phosphorylation (OXPHOS) for energy production and induction of cell death when converted into a non-specific channel (9). Opening MPTP decreases mitochondrial membrane potential (ΔΨm) and liberates pro-apoptotic factors, e.g., cytochrome c (Cyt c) and apoptosis inducing factor, triggering apoptosis (10). Formation and prolonged opening of the MPTP is the major event during freezing-induced injury leading to sudden collapse of mitochondrial function and disruption of energy supply, reducing motility, and fertilizing ability (11).

Melatonin (N-acetyl-5-methoxytryptamine) is synthesized from the essential dietary amino acid, tryptophan, in the pineal gland, and involved in the regulation of circadian and seasonal rhythms (12). It is a ubiquitous molecule found in a wide range of fluids, one of them being ram seminal plasma, in which it can reach higher concentrations than those found in blood, suggesting an extrapineal secretion by the testis (13, 14). Mitochondria are both a primary synthesis site and principle target for melatonin (15); this compound has high affinity for mitochondria and accumulates in these organelles, reversing mitochondrial dysfunction by reducing oxidative stress. Most of the beneficial effects of melatonin are related to its actions on mitochondrial physiology, including preventing mitochondrial impairment, energy failure and apoptosis in oxidatively-damaged mitochondria (5). In addition, melatonin also has an endocrine role in reproduction (16). Although melatonin protects sperm, including acrosome activity (17), membrane integrity (18), survival (19), and motility (20), little is known about direct effects of melatonin on cryopreservation-induced MPTP opening of mitochondria. We hypothesized that melatonin protects mitochondria of cryopreserved ram sperm by suppressing MPTP opening.

Our objective was to determine effects of melatonin on MPTP opening and sperm metabolism, including OXPHOS enzymes distribution, respiratory chain complex activity and oxygen consumption in frozen-thawed ram sperm.

## Methods

All procedures involving animals were approved by the Chinese Academy of Science Animal Care and Use Committee (Protocol No. 20120208). All chemicals were purchased from Sigma Aldrich (St. Louis, MO, USA), unless otherwise indicated.

### Overview of Experimental Design

**Figure 1:**
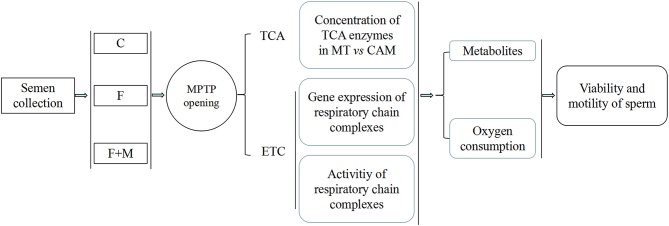
Experimental design for effects of melatonin on frozen-thawed sperm OXPHOS via regulating MPTP opening in ram. C was fresh sperm, F was frozen-thawed sperm, F+M (red column) was frozen-thawed sperm with melatonin. MT, mitochondria; CAM, cytoplasm.

Semen was collected, diluted in freezing solution (± melatonin) and subjected to slow freezing. First, effect of melatonin on MPTP were determined based on detection of opening state and sperm viability and motility. Then, to determine involvement of metabolism in protective effect of melatonin regulating MPTP opening, concentrations of key tricarboxylic acid cycle (TCA) enzymes in mitochondria and cytoplasm, gene expressions of mitochondrial coding respiratory chain complexes subunits and activity of complexes were assessed. Furthermore, oxygen consumption, adenosine triphosphate (ATP), lactate and acetyl-CoA contents were also measured to determine if melatonin improved OXPHOS (Figure 1).

### Rams and Diet

The experiment was conducted during the breeding season (August to October, i.e., late summer to late autumn) at the Changling Grassland Farming Ecological Experimental Station of Chinese Academy (44°33′N, 123°31′E). Ten Small-tail Han sheep rams (age, 2.5–3.0 y; body weight, 75–80 kg) were used. All rams had *ad libitum* access to water and to a 60:40 forage:concentrate ration (as recommended by the National Research Council).

### Semen Collection

Twice weekly for 10 wk, semen samples from each ram were collected using an artificial vagina (AV) containing water at 38–40°C. During each collection day, 10 rams were continuously collected twice, with a 30-min interval between collections (first ejaculates were discarded and second ejaculates were pooled). A graduated collecting tube attached to the disposable sleeve inside the AV was used to evaluate semen volume. Sperm concentration was calculated using a sperm density meter (SDM1, Minitube, Germany). Motility was estimated under 400 x magnification using a phase contrast microscope (Olympus BX60, Olympus, Japan). Ejaculates with volumes of 0.7–2.0 mL, concentrations >2.5 × 10^9^ sperm/mL and >80% motility were retained and pooled. A total of 20 pooled semen samples were used for subsequent analysis.

### Semen Dilution, Freezing, and Thawing

Basic semen extender (Tris-egg-yolk) contained an 80% (v:v) solution of 170 mM glucose, 104 mM sodium citrate, penicillin (10 IU/mL) and streptomycin (10 IU/mL) in distilled water and 20% (v:v) of egg yolk (fresh), whereas freezing solution contained 94% (v:v) of basic extender and 6% (v:v) glycerol. Freezing solution was supplemented with 0 (frozen group) or 10^−7^ M (frozen+melatonin group) of melatonin.

After each collection, pooled semen was incubated at 37°C in a water bath for 30 min, and then divided into multiple aliquots that were diluted to 1.0 × 10^9^ sperm/mL with corresponding freezing solutions. Diluted semen was chilled to 4°C over a 2-h interval, then kept at 4°C for 80 min (equilibration) and finally loaded into 0.25 mL straws. Filled straws were placed 4 cm above liquid nitrogen for 7 min and then plunged into liquid nitrogen. Samples were stored for at least 2 wk and subsequently thawed (40°C water bath for 15 s) and analyzed.

### MPTP Opening

The MPTP opening assay was an established calcein cobalt loading procedure, incubating sperm with calceinacetoxymethyl ester (Cal-AM), as described (21). Cal-AM is a fluorescent dye (1 kDa); the Cal-AM quenching method is a highly selective *in situ* indicator of sustained MPTP opening in mammalian cells. In the presence of CoCl_2_, Cal-AM fluorescence is quenched from the cytosol and nuclear compartments, but remains inside mitochondria. However, when the MPTP opens, Cal-AM fluorescence is lost from mitochondria. Briefly, fluorescence intensity (FI) of Cal-AM was measured to reflect MPTP opening. Motile sperm were loaded with Cal-AM (Dojindo, Kumamoto, Japan) to a final concentration of 15 mM in the presence of 30 mM CoCl_2_ and 0.1% Pluronic F127 in a dark chamber in a humidified incubator (37°C, 5% CO_2_ in air). After 20 min incubation, sperm were washed twice with phosphate buffered solution (PBS) to remove excess stain and quenching reagent. Samples were assayed within 2 h using a flow cytometer (Becton Dickinson, Sunnyvale, CA, USA). Data for 20,000 cells per sample were stored in the list mode using FACS Analyzer flow cytometry software (Becton Dickinson); thereafter, these data were passed through a Hewlett Packard computer (Palo Alto, CA, USA) with Consort 30 and analyzed by SuperCyt Analyst 3 software (Sierra Cytometry, Reno, NV, USA).

### ADP:ATP Ratio

The adenosine diphosphate (ADP):ATP ratio in sperm was measured with an EnzyLight^TM^ ADP:ATP Ratio Assay Kit (BioAssay Systems, Hayward, CA, USA), according to manufacturer's instructions. Firefly luciferase is a 62 KD monomer protein that catalyzes oxidation of ATP-dependent luciferase. Through electron transfer, chemical energy is converted into light energy by oxyluciferin, which is positively correlated with amount of ATP. The ratio of ADP:ATP was obtained by quantifying ATP concentration and then converted into ATP in the presence of pyruvate kinase (PK) to quantify ADP concentration. Briefly, 10 mL of washed sperm (1 × 10^6^) were mixed with 90 mL ATP reagent and vortexed for 30 s in a tube. After 1 min, the ATP relative light unit A (RLU A) was recorded and after 10 min, the reading was repeated to obtain ADP background RLU B (prior to measuring ADP). Immediately after reading RLU B, 5 ml ADP reagent was added to the tube and it was vortexed. After 1 min, ADP RLU C was read. We used this equation: ADP:ATP ratio = RLU C—RLU B/RLU A.

### Lactate and Acetyl-CoA Concentrations

Concentrations of lactate and acetyl-CoA concentrations were measured with ELISA kits (R&D Systems, Minneapolis, MN, USA), according to manufacturer's instructions. Briefly, sperm concentration was adjusted to 2 × 10^6^ /mL with PBS, centrifuged at 13,000 × g for 2 min, supernatant aspirated with a 1 mL needle tube and filtered with a 0.45 μm pore diameter fiber. Then, 100 μL of standard and sample were added to each well and the plate incubated for 2 h at 37°C. Liquid was removed and 100 μL of Biotin-antibody was added to each well and the plate incubated for 1 h at 37°C. Thereafter, each well was aspirated and washed (this process was done a total of three times). For this, each well was filled with 200 μL of Wash Buffer using a squirt bottle and after 2 min, all liquid was removed. After the last wash, residual Wash Buffer was removed by aspirating. Then, 100 μL of HRP-avidin were added to each well and the plate incubated for 1 h at 37°C. This aspiration and wash process was repeated five times. Thereafter, 90 μL of TMB Substrate were added to each well and plates were incubated for 30 min at 37°C (in the dark). After adding 50 μL of Stop Solution to each well, optical density of each well was determined within 5 min, using a microplate reader set to 450 nm. Absorbances were corrected by subtracting background reading. Concentrations of lactate and acetyl-CoA were calculated by interpolating values on a standard curve made for each plate. Each ELISA was performed in triplicate.

### Sperm Viability

Sperm concentration was adjusted to 1 × 10^6^/mL by addition of PBS. Then, 5 μL of 1.0 mM propidium iodide (PI) staining solution was added to diluted samples and they were incubated for 5 min in the dark. Thereafter, samples were centrifuged at 1,000 × g for 10 min, supernatant discarded and PBS added to adjust sperm concentration to 1 × 10^6^/mL. Samples were assayed within 2 h using a flow cytometer (Becton Dickinson, Sunnyvale, CA, USA). Red fluorescence emission for PI (i.e., 610/20 band pass) were measured in the FL2 filter. Two different populations can be recognized in this assay (Figure 3): viable sperm cells (M1: negative for PI) and dead sperm cells (M2: positive for PI). Data for 20,000 cells per sample were stored in the list mode using FACS Analyzer flow cytometry software (Becton Dickinson); thereafter, these data were passed through a Hewlett Packard computer (Palo Alto, CA, USA) with Consort 30 and analyzed with SuperCyt Analyst 3 software (Sierra Cytometry, Reno, NV, USA). Data were expressed as the percentage of fluorescent sperm. Each flow cytometer was performed in triplicate.

### Sperm Kinematics

For each sample of sperm, the kinematics was determined using a computer-aided sperm analysis system (Spermvision, Minitube, Tiefenbach, Germany) with the following settings: recording rate of 60 Hz, framers number of 30, minimum contrast of 60, minimum cell size of 10 pixels, low size gate of 0.60, high size gate of 8, low intensity gate of 0.25, high intensity gate of 1.50, non-motile head size of 5 pixels, non-motile head intensity of 55, progressive average path velocity (VAP) threshold of 50 mm/s, slow (static) cell VAP threshold of 10 mm/s, slow (static) cell straight line velocity (VSL) threshold of 0 mm/s, and straightness (STR) threshold of 80%. Cells exhibiting a VAP ≥ 50 mm/s and a STR ≥ 80% were considered progressive. Total motile sperm (TM), progressively motile sperm (PM), immotile (IM), curvilinear velocity (VCL), VAP, VSL, beat cross frequency (BCF), and average lateral (ALH) were assessed. In brief, sperm concentration was calculated using a sperm density meter (Minitube), diluted to 2.0 × 10^7^/mL in PBS and incubated in a water bath at 37°C for 5 min. Then, a 5-μL drop of sample was placed on a preheated glass slide (37°C). For each sample, five non-consecutive microscopic fields were randomly chosen on the slide and three slides per sample examined under 400 × magnification using a phase-contrast microscope (Axio Scope A1, Carl Zeiss, Oberkochen, Germany).

### Separation of Cytoplasm and Mitochondria

Sperm cytosolic and mitochondrial fractions were prepared with a Mitochondria/Cytosol Fractionation Kit (BioVision, Milpitas, CA, USA) according to manufacturer's instructions. Briefly, sperm were washed with 10 mL of ice-cold PBS and centrifuged at 600 × g for 5 min at 4°C. Supernatant was removed, sperm re-suspended in 1.0 mL of 1 × Cytosol Extraction Buffer Mix containing DTT and protease inhibitors and samples were incubated on ice for 10 min. Then, the sperm suspension was transferred to an ice-cold Dounce tissue grinder for 10 min, with sufficient strokes to effectively lyse cells. After microscopic examination to verify that homogenization was complete, homogenate was transferred to a 1.5 mL micro-centrifuge tube and centrifuged at 700 × g for 10 min at 4°C. Supernatant was collected into a fresh 1.5-mL tube and centrifuged at 10,000 × g for 30 min at 4°C. This supernatant was stored as the cytosolic fraction. The pellet was re-suspended in 0.1 mL Mitochondrial Extraction Buffer Mix containing DTT and protease inhibitors, vortexed for 10 s and saved as the mitochondrial fraction. To evaluate separation effect, a 5-μL drop of supernatant and mitochondrial suspension were each mixed with 0.2% Janus Green B dye solution of equal volume and dyed on slide for 3 min, rendering mitochondria blue-green when examined at 1000 X.

### OXPHOS Key Enzymes

Concentrations of Citrate synthase (CS), Isocitrate dehydrogenase (IDH), 2-Oxoglutarate dehydrogenase complex (OGDC) and F0F1-ATP synthase in cytosolic and mitochondrial fractions were measured with ELISA Kits (R&D systems, Minneapolis, MN, USA). Sperm samples were centrifuged at 1,000 × g for 5 min at 4°C. Re-suspended cells were collected and diluted with PBS (PH 7.0) and re-centrifuged at 1,000 × g for 5 min at 4°C. Collected samples were diluted with PBS (PH 7.0) to 1 × 10^9^/mL and stored overnight at −20°C. After two freeze-thaw cycles to rupture cell membranes, sperm lysates were centrifuged at 5,000 × g for 5 min at 4°C. Supernatant was collected and assayed immediately according to manufacturer's instructions. Each ELISA was performed in triplicate. Absorbances were corrected by subtracting background readings. Concentrations of key OXPHOS enzymes were calculated by interpolating values on a standard curve made for each plate.

### Respiratory Chain Complexes

Complex I (NADH- CoQ oxidoreductase), complex II (Succinate- CoQ oxidoreductase), complex III (CoQ- cytochrome c oxidoreductase) and complex IV (Cytochrome c oxidase) in mitochondrial fractions were measured with kits (Solarbio, Beijing, China), according to manufacturer's instructions. The reaction was assessed by following the decrease in absorbance with a diode-array spectrophotometer (PerkinElmer). Complex I catalyzes dehydrogenation of NADH to NAD^+^, with activity calculated by measuring (340 nm) rate of NADH oxidation. Reduced coenzyme Q, a catalytic product of complex II, can further reduce 2,6-dichloroindole phenol, which has a characteristic absorption peak at 605 nm. Activity of complex II can be calculated by measuring reduction rate of 2,6-dichloroindole phenol. Complex III has characteristic light absorption at 450 nm; therefore, an increasing rate of light absorption at 450 nm can reflect its activity. Complex IV catalyzes reductive cytochrome C to produce oxidized cytochrome C. Reduced cytochrome C has characteristic light absorption at 550 nm, so the rate of decrease in light absorption at 550 nm reflects activity of complex IV. Specific enzyme activity was expressed as: U/mg prot.

### Oxygen Consumption Rate (OCR) and Extracellular Acidification Rate (ECAR)

An XF24 extracellular flux analyzer (Seahorse Bioscience, North Billerica, MA, USA) was used to measure real time OCR and ECAR. The carbonylcyanidep-trifluo-romethoxyphenylhydrazone (FCCP group; an uncoupler of mitochondrial respiration) is a proton carrier, making many protons flow back and consuming much oxygen. However, this proton flow does not form ATP. Therefore, increased oxygen consumption after FCCP represents maximum oxygen consumption capacity of mitochondria (indirectly measuring maximum respiratory capacity), whereas the high value of its relative and basic value represents respiration potential. Finally, antimycin A and oligomycin were added; these respiratory chain inhibitors completely prevent mitochondrial oxygen consumption. Sperm concentration was adjusted to 2 × 10^6^ /mL and they were seeded on 20 wells of a XF24 plastic microplate coated with concanavalin A. However, four wells were left without cells (to determine background corrections). The plate was centrifuged for 2 min at 1,200 × g to ensure uniform adhesion of cells to the bottom of the well. The supernatant was removed and 500 μL of medium added to each well. All OCR and ECAR measurements were performed at 37°C under air. The OCR measurements were made from 42 to 90 min. After 54 min, 1 μM FCCP or mT medium (control group) were added to each well. Furthermore, 1 μM antimycin A and 1 μM rotenone were added to each well at 78 min after collection of samples. The OCR and ECAR values for each well were normalized by the number of sperm present and reported as: amol of O_2_ min^−1^ sperm^−1^ and nano-pH min^−1^ sperm^−1^. Basal mitochondrial OCR was calculated by subtracting OCR values obtained after addition of antimycin A and rotenone from OCR values at 54th min. Maximum mitochondrial OCR was calculated by subtracting OCR values obtained after addition of antimycin A and rotenone from OCR values recorded at 78th min in FCCP group. Spare OCR capacity was calculated by subtracting basal OCR values from maximum OCR values. Basal ECAR was calculated averaging ECAR values obtained from 42 to 63 min of the experiment.

### RNA Purification and qRT-PCR

Total RNA was extracted using TRIzol reagent (Invitrogen, Carlsbad, CA, USA) and RNase-free DNase used to remove genomic DNA. Integrity and concentration of RNA were determined by measuring absorbance at 260 nm. Total RNA (1.0 μg) from each sample was re-suspended in a 20 μL final volume of reaction buffer, containing 25 mM Tris-HCl, 37.5 mM KCl, 10 mM dithiothreitol, 1.5 mM MgCl_2_, 10 mM of each dNTP and 0.5 mg oligo (dT)_15_ primers to synthesize cDNA. After the reaction mixture reached 42°C, 20 units of reverse transcriptase was added to each tube and the sample incubated for 1 h at 42°C. Reverse transcription was stopped by denaturing the enzyme at 95°C. The final PCR mixture contained 2.5 μL cDNA, 1 × PCR buffer, 1.5 mM MgCl_2_, 200 μM dNTP mixture, 1 U of Taq DNA polymerase, 1 μM sense and antisense primers, and 5.0 μL sterile water. The qRT-PCR was conducted using the CFX96TM Real-Time PCR Detection System (Bio-Rad, Hercules, CA, USA) under standard conditions. Transcripts of mitochondrial coding complex I (MT-C I): *NADH dehydrogenase (ND)1, ND3, ND7*, complex III (MT-C III): *Cytochrome (Cyt) b*, complex IV (MT-C IV): *Cytochrome oxidase (COX) I, COX III*, and complex V (MT-C V): *ATPase (ATP) 6, ATP 8* were quantified in triplicate for each sample, with β-actin used as a reference. Expression levels were calculated using the comparative Ct (2^−ddCt^) method (22). Primers are listed in Table 1.

**Table 1:** Primer sequences and conditions.

**Gene**	**Primer sequence**	**T annealing (**°**C) x no. cycles**	**Fragment size (bp)**	**Accession number**
*ND1*	5′TAACATTGTTGGTCCATACG 3′ATGCTAGTGTGAGTGATAGG	58 × 35	91	NC_001941.1 (Oa)
*ND3*	5′GAAGCCAGGTCACCTTTCAA 3′TCCTGGAATCAACAAGCACA	57 × 35	76	NC_001941.1 (Oa)
*ND7*	5′TGCATGCTTCGAAACTCTGA 3′GGCCCCTAAACATTCCACTT	60 × 35	98	EU281461.1 (Bt)
*Cyt b*	5′CGGCTGACTAATCCGATACC 3′TGGGAGTACATAGCCCATGA	60 × 35	106	KT283254.1 (Oa)
*COX I*	5′GGGAGAAGCCTTAGTAGAGATTCTC 3′CGGGTGTCTACATCTAGGCCTACTGT	60 × 35	77	KT750038.1 (Oa)
*COX III*	5′CAGCCTAGTTCCTACCCACGAC 3′CCCGTTGCTATGAAGAATGTTG	59 × 35	103	EF490471.1 (Oa)
*ATP 6*	5′CGAACCTGAGCCCTAATA 3′GTAGCTCCTCCGATTAGA	59 × 35	84	NC_001941.1 (Oa)
*ATP 8*	5′CCTCAATGTCCCTGCTTCTG 3′GCCTCGCTGCTTTCTCTTG	58 × 35	114	NC_001941.1 (Oa)
*β-actin*	5′GTCATCACCATCGGCAATGA 3′CGTGAATGCCGCAGGATT	57 × 35	182	NM_001009784.3 (Oa)

*ND1, NADH dehydrogenase 1; ND3, NADH dehydrogenase 3; ND7, NADH dehydrogenase 7; Cyt b, cytochrome b; COX I, cytochrome oxidase I; COX III, cytochrome oxidase III; ATP 6, ATPase 6; ATP 8:ATPase 8; β-actin, beta-actin; Bt, Bos taurus; Oa, Ovis aries*.

### Statistical Analyses

Statistical analyses were performed using SPASS18.0 for Windows (SPSS Inc., Chicago, IL, USA). Initially, a Shapiro-Wilk test was done to test the hypothesis that numerical data were normally distributed. All data were expressed as mean ± SD and were analyzed by analysis of variance (ANOVA), with *P*<*0.05* considered significant and Duncan's multiple range test used to locate differences. The homogeneity of variances was checked through Levene test.

## Results

### Effects of Melatonin on MPTP Opening, Viability, Motility, Pyruvate Metabolism and ATP Production

**Figure 2:**
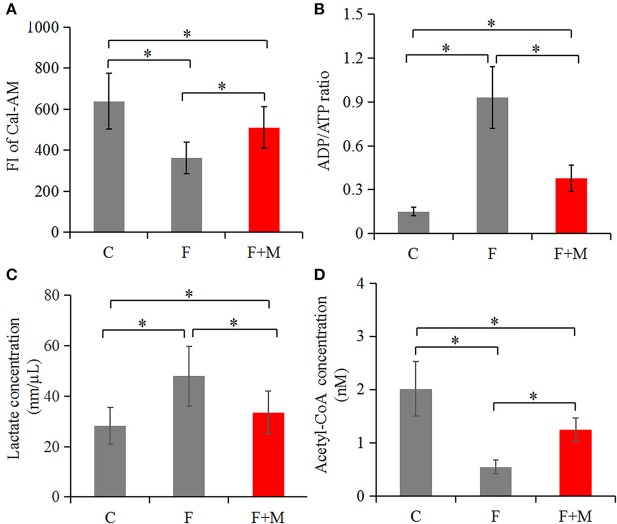
Concentrations of Cal-AM **(A)**, ADP/ATP **(B)**, lactate **(C)**, and acetyl-CoA **(D)** in fresh, frozen and melatonin-treated ram sperm (*n* = 10). C was fresh sperm, F was frozen-thawed sperm, F+M (red column) was frozen-thawed sperm with melatonin. The MPTP opening assay was performed via Cal-AM fluorescent staining with flow cytometry analysis; ADP/ATP, lactate and acetyl-CoA were measured with related assay Kit using a microplate reader. The experiments were repeated three times. *P < 0.05.

**Figure 3:**
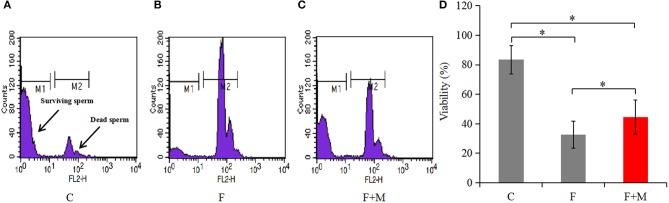
Viability of fresh, frozen, and melatonin-treated ram sperm (*n* = 10). C was fresh sperm, F was frozen-thawed sperm, F+M (red column) was frozen-thawed sperm with melatonin. Viability was determined by PI staining with flow cytometry **(A–C)**, the percentage of fluorescent sperm in of fresh, frozen, and melatonin-treated ram sperm **(D)**. The experiment was repeated three times. *P < 0.05.

**Figure 4:**
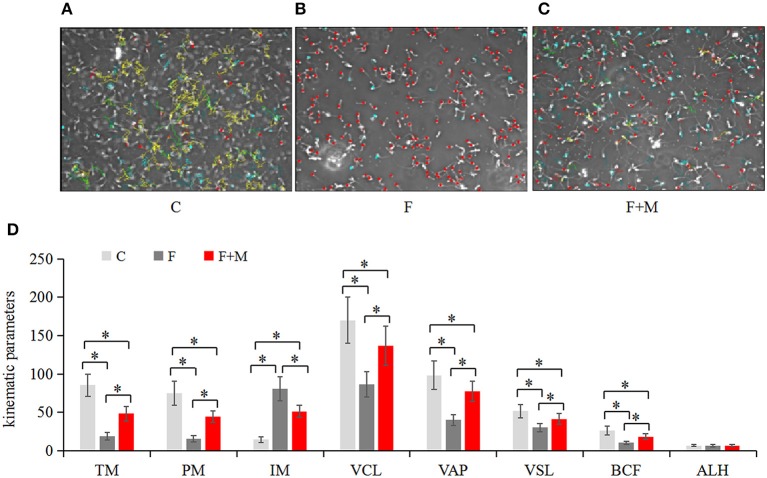
Motility of fresh, frozen and melatonin-treated ram sperm (*n* = 10). C was fresh sperm, F was frozen-thawed sperm, F+M (red column) was frozen-thawed sperm with melatonin. Motility was determined with a computer-aided sperm analysis system **(A–C)**, the kinematic parameters in fresh, frozen and melatonin-treated ram sperm **(D)**. TM, total motile sperm (%); PM, progressive motile sperm (%); IM, immotile (%); VCL, curvilinear velocity (μm/sec); VAP, average path velocity (μm/sec); VSL, straight line velocity (μm/sec); BCF, beat cross frequency (hertz); ALH, average lateral (μm). Green represented progressive sperm, red represented immotile sperm and blue represented motile (but not progressive) sperm **(A–C)**. The experiment was repeated three times. *P < 0.05.

In the melatonin group, FI of Cal-AM was higher compared to those of the frozen group, but lower compared to the control group (*P* < 0.05, Figure 2A); therefore, melatonin effectively inhibited cryo-induced MPTP opening. In the melatonin group, glucose metabolite concentrations were physiological and concentrations of acetyl-CoA were higher compared to those of the frozen group, but lower compared to the control group (*P* < 0.05, Figure 2D); however, lactate concentrations were lower compared to those of the frozen group, but higher compared to the control group (*P* < 0.05, Figure 2C). Cryopreservation inhibited OXPHOS and enhancement of glycolysis, whereas melatonin counteracted respiratory chain inhibition. We speculated that melatonin may have increased ATP production and sperm quality in cryopreservation-induced mitochondrial dysfunction. Therefore, by flow cytometry and CASA analysis, melatonin also increased (*P* < 0.05) viability (Figure 3) and motility (TM, PM, IM, VCL, VAP, VSL, and BCF; Figure 4) of frozen-thawed sperm. Concurrently, there was a lower *(P* < 0.05) ADP:ATP ratio in frozen-thawed sperm after melatonin treatment (Figure 2B), indicating that melatonin not only improved sperm ATP production, but also inhibited sperm apoptosis.

### Effects of Melatonin on Key OXPHOS Enzymes

**Figure 5:**
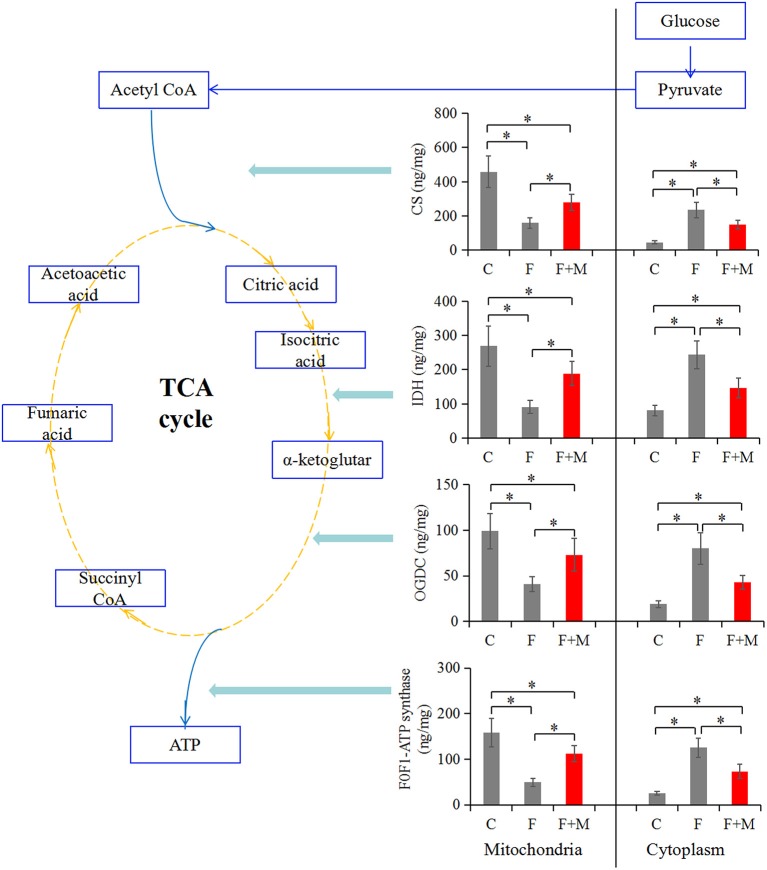
Concentrations of CS, IDH, OGDC, and F0F1-ATP synthase in fresh, frozen, and melatonin-treated ram sperm (*n* = 10). C was fresh sperm, F was frozen-thawed sperm, F+M (red column) was frozen-thawed sperm with melatonin. TCA, tricarboxylic acid cycle; CS, citrate synthase; IDH, isocitrate dehydrogenase; OGDC, 2-Oxoglutarate dehydrogenase complex. The experiment was repeated three times. *P < 0.05.

Improved OXPHOS in frozen-thawed sperm supplemented with melatonin may be driven by enzymatic reactions. Therefore, to clarify the relationship between MPTP and improved OXPHOS, concentrations of the TCA cycle enzymes were analyzed in mitochondrial and cytosolic fractions. In the melatonin group, concentrations of CS, IDH, OGDC and F0F1-ATP synthase in the mitochondrial fraction were higher (*P* < 0.05) compared to those of the frozen group, but lower compared to the control group (*P* < 0.05, Figure 5). Furthermore, in the cytosolic fraction, they were lower (*P* < 0.05) compared to those of the frozen group, but higher compared to the control group (*P* < 0.05, Figure 5). Melatonin reduced permeability of CS, IDH, OGDC and F0F1-ATP synthase, keeping them from moving from mitochondrial to cytosolic fractions, by suppressing cryopreservation-induced MPTP opening.

### Effects of Melatonin on mRNA Expression and Activity of Respiratory Chain Complexes

**Figure 6:**
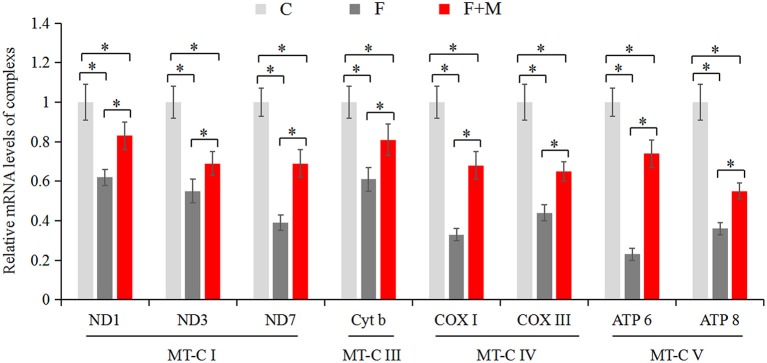
Gene expressions of mitochondria encode respiratory chain complexes in fresh, frozen, and melatonin-treated ram sperm (*n* = 10). C was fresh sperm, F was frozen-thawed sperm, F+M (red column) was frozen-thawed sperm with melatonin. MT-C I, mitochondrial coding complex I; MT-C III, mitochondrial coding complex III; MT-C IV, mitochondrial coding complex IV; MT-C V, mitochondrial coding complex V. The experiment was repeated three times. *P < 0.05.

The mitochondrial electron transport chain (ETC) leads to ATP synthesis through the OXPHOS pathway. Expression and activity of respiratory chain complexes determined efficiency of ATP synthesis. As mature sperm are unable to synthesize nuclear genome encoded new proteins, we only detected mitochondrial genome encoded complex subunits by real-time quantitative PCR. In the melatonin group, mRNA expression of *ND1, ND3, ND7, Cyt b, COX I, COX III, ATP 6*, and *ATP 8* (Figure 6) were higher (*P* < 0.05) compared to those of the frozen group, but lower compared to the control group (*P* < 0.05). In addition, there were higher activities (*P* < 0.05) of complex I, II, III, and IV (Table 2) in frozen-thawed sperm after melatonin treatment.

**Table 2:** Activities (U/mg prot) of respiratory chain complexes in fresh, frozen and melatonin-treated ram sperm.

**Group**	**Complex I**	**Complex II**	**Complex III**	**Complex IV**
Control	449 ± 101.3^a^	65.6 ± 14.2^a^	19.3 ± 5.09^a^	98.5 ± 19.4^a^
Frozen	183 ± 34.1^c^	25.1 ± 6.14^c^	8.77 ± 2.13^c^	51.4 ± 12.1^c^
Frozen+Melatonin	314 ± 58.7^b^	44.5 ± 7.33^b^	13.4 ± 2.38^b^	66.3 ± 13.7^b^

*Control was fresh semen*.

*The experiment was repeated three times*.

a−c *Within a column, means without a common superscript differed (P < 0.05)*.

### Effects of Melatonin on Oxygen Consumption

**Figure 7:**
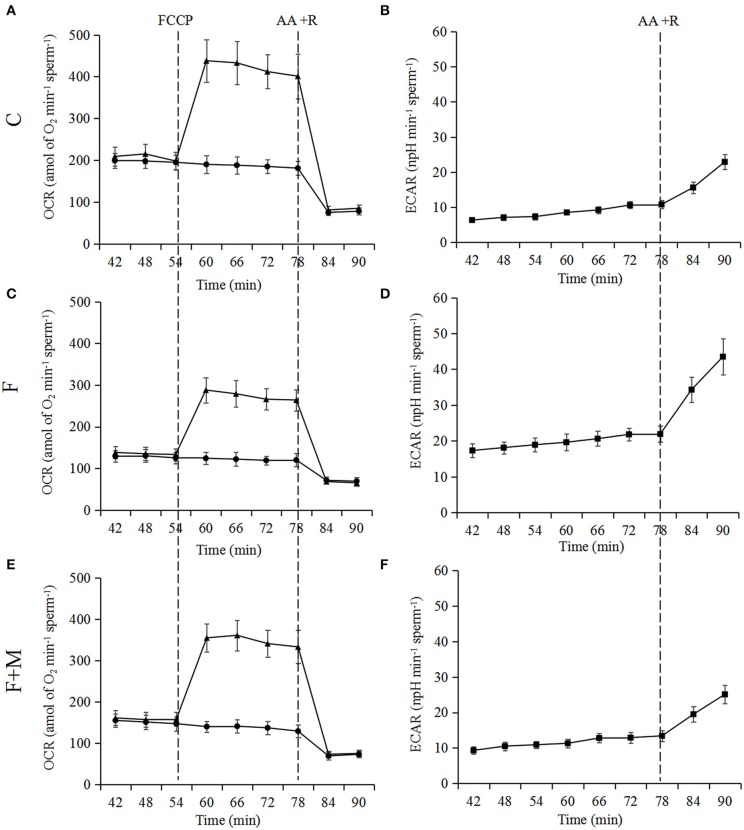
Test of OCR **(A,C,E)** and ECAR **(B,D,F)** in fresh, frozen, and melatonin-treated ram sperm (*n* = 10). C was fresh sperm, F was frozen-thawed sperm, F+M (red column) was frozen-thawed sperm with melatonin. OCR, oxygen consumption rate; ECAR, extracellular acidification rate. Treatments were as follows: control (black dots) and FCCP (black triangles). Additions were indicated with a dotted line. Final concentrations of FCCP, antimycin A and rotenone were all 1 μM.

**Figure 8:**
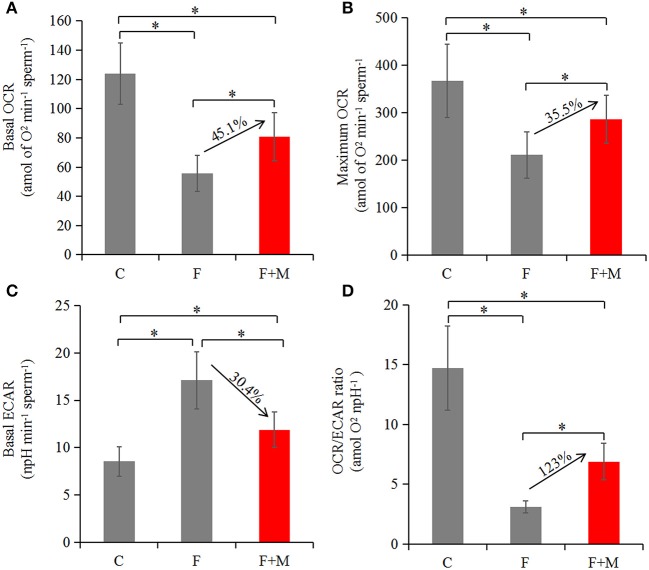
Oxygen consumption in fresh, frozen and melatonin-treated ram sperm (*n* = 10). C was fresh sperm, F was frozen-thawed sperm, F+M (red column) was frozen-thawed sperm with melatonin. Basal OCR **(A)**, maximum OCR **(B)**, basal ECAR **(C)**, and OCR/ECAR **(D)** in fresh, frozen and melatonin-treated sperm. The experiment was repeated three times. *P < 0.05.

Based on optimizing distribution of key TCA enzymes inside and outside mitochondria and increasing activity and expression of respiratory chain complexes, we analyzed sperm oxygen consumption to further assess respiratory efficiency of mitochondria in frozen-thawed sperm. In the melatonin group, basal OCR, maximum OCR and OCR/ECAR were 45.1, 35.5, and 123% higher (*P* < 0.05; Figures 7, 8A,B,D), respectively, whereas basal ECAR was 30.4% lower compared to the frozen group (*P* < 0.05; Figures 7, 8C).

## Discussion

Bioenergetic functions of sperm mitochondria modulate ATP formation through the mitochondrial ETC, leading to ATP synthesis through the OXPHOS pathway, critical to sustaining sperm viability and motility (23). In human (24) and animal (25, 26) sperm, freezing causes damage, decreasing both motility and fertilizing potential. However, some protective effects of melatonin were reported at mitochondrial level (27). In this study, suppression of MPTP opening, improvements in key OXPHOS enzymes and oxygen consumption, indicated that melatonin protected mitochondria from cryoinjury and promoted ATP synthesis of frozen-thawed ram sperm.

Mitochondria are the sperm structure most sensitive to cryopreservation, manifested as changes in metabolism (28). Increasing activities of total ATPase, creatine kinase, succinate dehydrogenase, lactate dehydrogenase, superoxide dismutase, catalase, and glutathione peroxidase in seminal plasma, but decreasing activities in frozen-thawed sperm, indicated that freezing damaged mitochondrial membrane permeability (29). Permeability transition was attributed to opening of the MPTP, implicated as a major cause of abnormal mitochondrial structure and function (30). In this study, addition of melatonin in freezing solution profoundly inhibited cryopreservation-inducing MPTP opening; this was attributed to inhibition of PPIase activity and Ser 31phosphorylation of cyclophilin D (Cyp D) and decreases in Cyp D and adenine nucleotide translocator (ANT) activities (31, 32). Opening of MPTP causes release of pro-apoptotic factors into the cytoplasm, promoting cell degradation. In this study, based on increasing viability and motility of sperm, we inferred that melatonin inhibited apoptosis and improved quality of frozen-thawed sperm. Inhibition of the opening of MPTP during cryopreservation significantly reduced apoptotic markers (e.g., caspase activity) and mitochondrial membrane permeability in equine sperm (33); furthermore, it also increased ΔΨm and improved fertilization in frozen-thawed sea urchin sperm (34). Therefore, results of the current study confirmed that melatonin effectively prevented cryopreservation-induced MPTP opening in ram sperm.

OXPHOS, a major mitochondrial reaction, is more efficient than glycolysis for generating ATP. The TCA cycle is responsible for providing electrons necessary for ETC. Unlike sperm from other mammals [e.g., mice; (35)], OXPHOS apparently has a more important role than glycolysis in supplying energy for motility in sheep sperm. Many key enzymes, e.g., succinate dehydrogenase complex A, had a significant genotype association with impaired sperm production, leading to mitochondrial respiratory chain activity impairment (36). In our study, cryopreservation not only induced CS, IDH, OGDC, and F0F1-ATP synthase permeability from mitochondrial to cytosolic fractions, but also decreased activities of respiratory chain complexes. Similar to our study, cryopreservation decreased activities of complexes II and IV and reduced ATP production in fish sperm (37). Therefore, we inferred that inhibiting the mentioned complexes induced by cryopreservation contributed to sperm metabolic disorders. However, based on increasing concentrations of key TCA enzymes in mitochondria and activities of complexes in the respiratory chain in this study, adding melatonin before freezing enhanced the OXPHOS process, which was closely related to melatonin-inhibited opening of MPTP, as opening of MPTP not only increased mitochondrial permeability transition (38), but also inhibited mitochondrial complexes (30).

**Figure 9:**
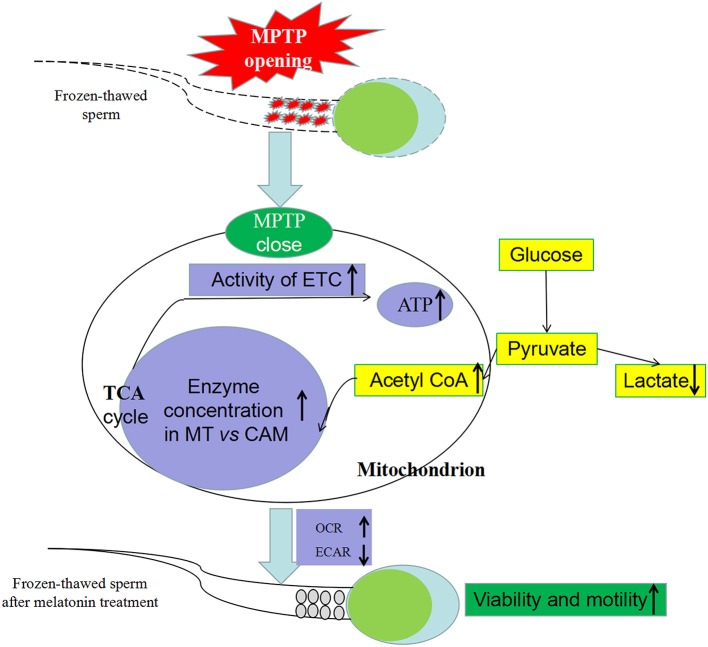
Effect of melatonin on frozen-thawed sperm metabolism by regulating MPTP opening. MPTP: mitochondrial permeability transition pore, ETC: mitochondrial electron transport chain, OCR: oxygen consumption rate, ECAR, extracellular acidification rate; MT, mitochondria; CAM, cytoplasm.

In various studies, high-dose exogenous melatonin enhanced mitochondrial energy homeostasis (39–41). Furthermore, melatonin alleviated inhibitory effects of complexes I and IV (42) and increased ATPase activity (42, 43) in numerous metabolic disorders and various cellular models. Increasing mRNA expression and activity of complexes in our study indicated that melatonin counteracted respiratory chain inhibition and may increase ATP production in cryopreservation-induced mitochondrial dysfunction. Moreover, based on sperm oxygen consumption, we inferred that melatonin significantly improved mitochondrial respiratory capacity and the OXPHOS pathway produced greater amounts of ATP, thereby improving kinetics of frozen-thawed sperm, as motility strongly depends on mitochondrial ATP and is positively correlated with mitochondrial-encoded complex activities (44) and oxygen consumption (45). Therefore, suppression of cryopreservation-induced MPTP opening by melatonin was linked to improved sperm metabolism by OXPHOS (Figure 9).

Based on lower basal OCR and acetyl-CoA concentration and higher basal ECAR and lactate concentration of frozen-thawed sperm, freezing led to conversion of sperm-dependent metabolic pathways, mainly manifested in inhibition of oxidative phosphorylation and enhancement of glycolysis. This may be a self-protective mechanism of sperm regulating glycometabolism, with conversion from energy-consuming metabolism to protective and reparative metabolism (46, 47). MPTP opening may trigger a glycolysis/oxidative phosphorylation mode transition in frozen-thawed sperm. However, based on higher basal OCR and acetyl-CoA concentration and lower basal ECAR and lactate concentration, we inferred that melatonin can correct abnormal metabolic patterns to alleviate sperm metabolic disorders, possibly by inhibiting MPTP opening.

In summary, mechanisms of the protective effects of the melatonin on mitochondria included: (1) optimizing distribution of OXPHOS enzymes in sperm and (2) increasing activities of respiratory complexes. Consequently, melatonin not only reduced MPTP opening, but also improved mitochondrial metabolism in frozen-thawed ram sperm. Therefore, melatonin was an effective cryo-protectant; it improved OXPHOS to ATP production by optimizing distribution of TCA key enzymes in mitochondria and cytoplasm and by increasing activity of ETC in frozen-thawed sperm. Furthermore, it improved sperm viability and motility, attributed to inhibition of cryopreservation-induced MPTP opening. This study yielded new knowledge regarding cryopreservation-induced reductions in ram sperm quality, and may provide a basis for novel, evidence-based approaches to improve quality of frozen-thawed ram sperm.

## Data Availability Statement

The datasets generated for this study are available on request to the corresponding author.

## Ethics Statement

All procedures involving animals were approved by the Chinese Academy of Science Animal Care and Use Committee (Protocol No. 20120208).

## Author Contributions

YF and RZ conceived and designed experiments and wrote the paper. YF, CZ, HX, and XZ performed experiments. YF and CZ analyzed data. YF, HX, and XZ contributed reagents, materials, and analysis tools. All authors contributed to interpretation of data and reviewed the manuscript.

### Conflict of Interest

The authors declare that the research was conducted in the absence of any commercial or financial relationships that could be construed as a potential conflict of interest.
